# Temporomandibular Joint Gap Arthroplasty in Cats

**DOI:** 10.3389/fvets.2020.00482

**Published:** 2020-08-13

**Authors:** Armeti Aghashani, Frank J. M. Verstraete, Boaz Arzi

**Affiliations:** ^1^William R. Pritchard Veterinary Medical Teaching Hospital, School of Veterinary Medicine, University of California, Davis, Davis, CA, United States; ^2^Department of Surgical and Radiological Sciences, School of Veterinary Medicine, University of California, Davis, Davis, CA, United States

**Keywords:** temporomandibular joint, ankylosis, pseudoankylosis, cat, gap arthroplasty, piezosurgery, cone-beam computed tomography

## Abstract

Temporomandibular joint (TMJ) ankylosis is defined as fibrous or bony fusion of the mandibular head of the condylar process and the mandibular fossa of the squamous part of the temporal bone. Ankylosis of the TMJ may be intraarticular, extraarticular, or both. The objective of this report is to describe the surgical planning, technique, and outcome of gap arthroplasty for extensive TMJ ankylosis in cats. Client-owned cats (*n* = 7) were examined clinically and surgical planning included the use of cone-beam computed tomography (CBCT) and tridimensional (3D) printed models. In six of the seven cats, temporary tracheostomy intubation was required. Gap arthroplasty included zygomectomy, coronoidectomy, condylectomy, as well as fossectomy (removal of the mandibular fossa of the temporal bone) and was performed using a piezosurgical unit. In all seven cats, gap arthroplasty was performed without surgical complications. In addition, a clinically acceptable mouth opening was achieved in all cases. However, a noticeable mandibular instability was observed. Medium-term follow-up demonstrated acceptable quality of life with one case of recurrence of ankylosis requiring repeated bilateral surgery, and a second case with recurrence of ankylosis not requiring surgical intervention at the time of manuscript preparation. We concluded that TMJ gap arthroplasty in cats is a salvage procedure indicated in cases of severe intraarticular and extraarticular ankylosis. Diagnostic imaging by means of CBCT and 3D printing are essential for precise surgical planning. The use of a piezosurgical unit allows for safe and precise ostectomy. Clinically, despite the resulting mandibular instability, appropriate prehension of food and water was possible.

## Introduction

Temporomandibular joint (TMJ) ankylosis and pseudoankylosis is an uncommon but severely debilitating condition that can be seen in various species (1–4). A rapidly progressive inability to open the mouth is most commonly noted, often with a history of maxillofacial trauma (5). Ankylosis of the TMJs prevents basic and important functions such as prehension of food, grooming, vocalization, thermoregulation, and adequate water intake. Additional implications of TMJ ankylosis may be skeletal and/or dental malocclusion, periodontitis, and mucosal ulceration (2, 6–8). Cats with TMJ ankylosis may be presented on an urgent or emergent basis due to tongue entrapment and secondary swelling, which can result in distress and death.

TMJ ankylosis is defined as fibrous or bony fusion of the mandibular head of the condylar process and the mandibular fossa of the squamous part of the temporal bone. Ankylosis can be further defined as intraarticular, extraarticular, or a combination of both (4, 6, 9). Intraarticular ankylosis involves destruction of the fibrocartilaginous disc, narrowing of the joint space, and flattening of the mandibular head (9). Extraarticular ankylosis occurs due to encapsulation of the joint or structures near the joint (i.e., the zygomatic arch and coronoid process of the mandible) with little or no involvement within the joint (5, 9). Extraarticular ankylosis may be termed “pseudoankylosis” due to mechanical obstruction or fusion of bones surrounding the joint (4, 6). Often times, severe cases have a combination of intraarticular and extraarticular ankylosis (9).

Cats most commonly are presented at a young age with a known history of maxillofacial trauma, typically secondary to falling, having been bitten by a dog, or vehicular trauma (10, 11). If there is no known history of trauma, diagnostic imaging is often suggestive of historic trauma. Other differential diagnoses include infection (i.e., osteomyelitis or retrobulbar disease), a congenital malformation, or less likely, neoplasia (1, 2, 6–8, 12, 13). Depending on age at the time of trauma, there may be discordant maxillofacial growth or abnormal tooth eruption (1, 4, 10). Oral examination of these cats is often unrewarding due to the limited ability to open the mouth. In some cases, proliferative abnormal bone growth may be palpable in the region of the TMJs. Close attention should be paid to the actual range of motion of the jaws as this will likely to have implications for future anesthesia and surgical treatment of this condition.

The accurate and precise diagnosis of lesions of the TMJ requires the use of advanced imaging. Computed tomography (CT) and cone-beam CT (CBCT) are considered the gold-standard for imaging for the TMJ (1, 3, 14). The use of CT images with 3D reconstruction is a helpful tool to aid in anatomic understanding and surgical planning (4, 15). In addition, the use of 3D printed skulls has been advocated as a surgical planning tool and for reference in the operating room (4, 15).

This retrospective study describes the clinical features, surgical treatment, and outcome of a gap arthroplasty procedure in a series of seven cats affected by extensive intraarticular and/or extraarticular TMJ ankylosis. We further aim to exemplify the use of 3D printing and piezosurgical technique for precise surgical planning and execution.

## Materials and Methods

### Case Inclusion

Seven client-owned cats were presented to the Dentistry and Oral Surgery Service at the William R. Pritchard Veterinary Medical Teaching Hospital, University of California, Davis, for assessment and treatment of intraarticular and/or extraarticular TMJ ankylosis between September 2017 and December 2019. The cats were between 5 and 18 months old (mean age of 7.7 months). All cats experienced a severe restriction in opening of the mouth which was subsequently treated with gap arthroplasty (Table 1).

**Table 1:** Case information, TMJ involvement, complications, and follow-up (including recheck appointments with or without diagnostic imaging).

**Cat**	**Age (months)**	**Sex**	**Weight at diagnosis (kg)**	**Known history of trauma**	**Unilateral or bilateral treatment**	**Complications with surgery or during recovery**	**Duration of Follow-up (months)**
1	6	F	3.3 kg	High-rise fall	Bilateral	Fracture line into the calvarium with gas tracking intracranially	13
						Postoperatively developed left mandibular drift and malocclusion	
2	18	FS	2.7 kg	Dog bite injury	Bilateral (staged surgery right side first)	6-months postoperative left-sided pseudojoint formation with substantial narrowing of gap	10
3	5	F	2.0 kg	Hit by car	Bilateral	Lack of blink of the left eye	3 (by referring veterinarian)
4	7	FS	3.0 kg	Unknown	Bilateral	A small portion of the left retroarticular process remaining postoperatively	6
5	5	MC	3.4 kg	Unknown	Bilateral	None	4
6	6	F	2.1 kg	Historic jaw fracture (unknown etiology)	Unilateral (left sided surgery)	Persistent malocclusion postoperatively with secondary trauma	2
7	7	MC	2.7 kg	Unknown	Bilateral (for first and second surgery)	Left-sided incision partial dehiscence 10-days postoperatively with resolving subcutaneous emphysema	4
						Suture removal for second surgery—cat had ~50% of normal range of motion	

### Medical Records Review

Information was obtained from the medical records including history, physical examination, laboratory diagnostics, diagnostic imaging results, surgical treatment, and outcome, along with follow-up care. CBCT images were reviewed and 3D printed skulls were examined for all included cases. Follow-up was obtained from medical records and telephone interviews with owners.

### Cone-Beam Computed Tomography (CBCT)

Preoperative CBCT scans were obtained to allow for the evaluation of anatomical and structural features of the TMJs along with the rest of the skull and dentition. Six of seven cats were heavily sedated while the last cat was anesthetized for preoperative CBCT scans (Table 2). The TMJs were evaluated using a CBCT unit (NewTom 5G CBCT scanner, NewTom, Verona Italy). The field of view was 15 × 12 cm, and serial sections of the skull were obtained with a scan time of 18 s, which resulted in a voxel size (slice thickness) of 150 μm. The images were evaluated with 3D rendering using Invivo5 software (Anatomage, San Jose, CA) by two experienced board-certified veterinary dentists and maxillofacial surgeons (BA and FV) and a board-certified radiologist. The location and extent of the anatomic changes associated with the intraarticular and/or extraarticular ankylosis were documented.

**Table 2:** Sedation and anesthetic protocol.

**Case**	**Diagnostic imaging**	**Surgical anesthetic protocol**	**Temporary tracheostomy**	**Suture removal**
1	Heavy sedation with ketamine, midazolam, and dexmedetomidine. No intubation performed	Ketamine, midazolam, and dexmedetomidine premedication. Induction with alfaxalone	Yes, no complications	Sedation with dexmedetomidine IM
2	Heavy sedation with butorphanol, midazolam, and dexmedetomidine. No intubation performed	Methadone and atropine premedication. Induction with ketamine and midazolam (same protocol for both surgeries)	Yes (right side), no complications	No sedation needed
			Second surgery: normal intubation for the left side.	
3	Heavy sedation with midazolam and dexmedetomidine with intermittent propofol administration. No intubation performed	Methadone premedication. Induction with ketamine and midazolam	Yes, no complications	Not performed at UC Davis
4	Heavy sedation with alfaxalone, butorphanol, and midazolam. No intubation performed	Methadone, atropine, and dexmedetomidine premedication. Induction with ketamine and midazolam	Yes, no complications	Not performed at UC Davis
5	Anesthesia with oral intubation using a stylet. Premedication with butorphanol and atropine. Induction with alfaxalone and midazolam	Alfaxalone, hydromorphone, atropine premedication. Induction with alfaxalone and midazolam	No, endotracheal tube passed using a stylet	No sedation needed
6	Heavy sedation with butorphanol and dexmedetomidine. No intubation performed	Dexmedetomidine and hydromorphone premedication. Induction with ketamine and midazolam	Yes, no complications	No sedation needed
7	Heavy sedation with dexmedetomidine. No intubation performed	Methadone and dexmedetomidine premedication. Induction with ketamine and midazolam (same protocol for both surgeries)	Yes, no complications	Sedation with gabapentin orally for both suture removal appointments
			Second surgery: endotracheal tube passed via endoscopic guided stylet placement	

*In all cases standard drug doses were used.

### 3D Model Printing

**Figure 1:**
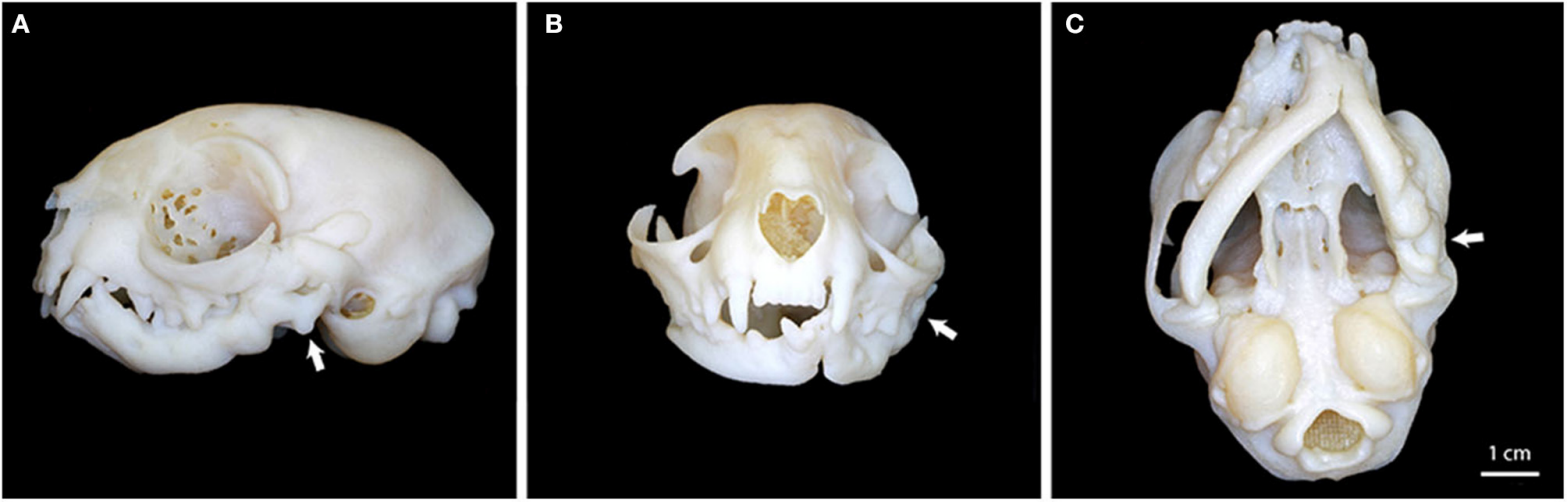
Three-dimensional printed model of the cat depicted in Figure 3. **(A)** Left lateral view depicting ankylosis of the left TMJ (white arrow). **(B)** Significant asymmetry and malocclusion of the mandibles, along with left TMJ ankylosis (white arrow). **(C)** Ventral view depicting mandibular asymmetry and the region of ankylosis (white arrow).

For initial surgeries in all cats, 3D volume renderings, and 3D printing of the CBCT images were created for surgical planning purposes as previously described (15). The CBCT was compiled into a Standard Tessellation Language mesh. A 3D model was then printed to scale using an Object Connex 260V Polyjet Printer (Object/Stratasys, Rehovot, Israel). The 3D model was used to mark the sites of planned osteotomy prior to surgery and as a visual aid during surgery (Figures 1A–C).

### Anesthesia

All cats were placed under general anesthesia for gap arthroplasty using variable anesthetic protocols as described in Table 2. All cats were given anti-emetics prior to anesthesia as cats with limited opening of the mouth are prone to aspiration pneumonia. Temporary tracheostomy was performed without complication in six of the seven cats as described elsewhere (16–18). In the cat with staged surgery, a temporary tracheostomy for the first surgery and a routine oral intubation for the second surgery were performed. In the cat with recurrent ankylosis, the cat had a temporary tracheostomy for the first surgery and endoscopic-guided endotracheal tube placement for the second surgery. In one cat, the mouth was able to open wide enough for a stylet to be guided down the airway and an endotracheal tube to be passed over the stylet.

### Surgical Technique

**Figure 2:**
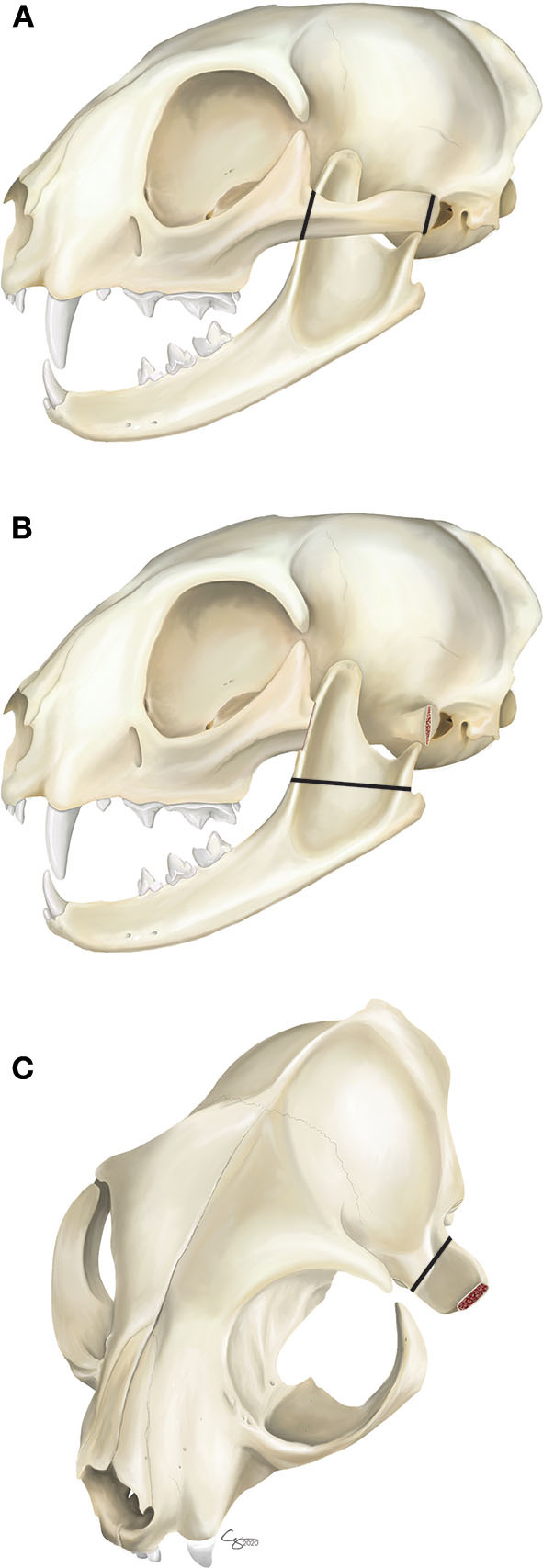
Gap arthroplasty surgical ostectomies, **(A)** The first ostectomies are made on the zygomatic arch caudal to the orbital ligament and just rostral to the TMJ (i.e., zygomectomy). **(B)** The second ostectomy involves removal of the coronoid process and condylar process just above the level of the mandibular foramen. **(C)** The third ostectomy is made on the medial aspect of the mandibular fossa of the squamous part of the temporal bone (i.e., fossectomy).

All affected cats were clipped on both sides of the head and aseptically prepared for surgery. The cats were placed in lateral recumbency with the face and cervical region slightly elevated. A lateral approach to the TMJ area was performed as recently described (6). Briefly, a full-thickness incision along the length of the zygoma on the ventral aspect was performed. A periosteal elevator was used to elevate the periosteum from the zygomatic arch, coronoid process, condylar process, and around the mandibular fossa of the squamous part of the temporal bone. In all cases, a piezosurgical unit (Implant Center 2® or Piezotome® Cube, Acteon, Mérignac, France) with a bone cutting (osteotomy) tip (BS1S or BS1L Acteon, Mérignac, France) was used to perform the ostectomies, avoiding damage to blood vessels and nerves (19, 20). The first ostectomy involved removal of the majority of the zygomatic arch (i.e., zygomectomy) just caudal to the orbital ligament, and extending up to the mandibular fossa (Figure 2A). This allowed for exposure of the caudal mandible in preparation for the second ostectomy. The second ostectomy involved removal of the coronoid process and condylar process just above the level of the mandibular foramen (Figure 2B). The aim of the second ostectomy was to cut rostrally in a direct line to avoid the mandibular foramen and associated neurovascular bundle. The third ostectomy was made on the medial aspect of the mandibular fossa of the squamous part of the temporal bone (i.e., fossectomy; Figure 2C). This final ostectomy was made at the level of the retroarticular process, with care to direct the piezotome away from the calvarium.

Once the cut bones were removed, the bone edges were smoothed as needed with the piezotome using a curved scalpel tip (BS6 Acteon, Mérignac, France). The surgical site was flushed with sterile saline and a 3-layer closure was performed. The drapes were removed, and the range of motion of the jaws was evaluated and measured. If disease was unilateral and the cat had normal range of motion, the decision was made to stop with unilateral surgery. If range of motion was inhibited or restricted the cat was turned and the same procedure was performed on the other side for bilateral surgical treatment.

### Postoperative Imaging

All cats had immediate postoperative CBCT performed to assess the ostectomies and to document that an appropriately sized gap had been made between the cut segments of bone. All postoperative imaging was performed with the cat's mouth in an open position to evaluate and document the mouth opening (i.e., immediate surgical outcome).

### Postoperative Considerations

Cats with a temporary tracheostomy were recovered in an intensive care unit to allow for safe extubation and monitoring of respiratory status upon anesthetic recovery. The tracheostomy sites were left to heal by second intention without the placement of sutures or neck bandages.

IV fluid therapy was continued postoperatively until the cats were eating independently. Pain management was achieved using a multimodal approach. Postoperative pain was assessed using the Colorado State University Feline Acute Pain Scale (a 4-point scale). Non-steroidal anti-inflammatory drugs were administered via injection or by mouth for 1–3 days duration. Injectable opioid pain medications were administered in the first 8–12 hours postoperatively. The cats were then transitioned onto a transmucosal pain management with buprenorphine for an additional 5–7 days. If additional pain control or sedation was needed, the cat was also prescribed gabapentin. All cats were discharged with an Elizabethan collar and skin sutures that required removal in 10–14 days. All cats were also discharged with a 10–14 day course of postoperative antibiotics (amoxicillin trihydrate/clavulanate potassium).

## Results

On initial presentation, all cats were slightly underweight with unkempt fur but otherwise in good physical condition. Only one cat was estimated to be ~5% dehydrated on initial physical examination. The results of hematological, and serum biochemical analysis were considered within normal limits for young cats. All cats were blood-typed and cross-matched in preparation for surgery (5 type A and 2 type B).

### Mouth Opening

**Figure 3:**
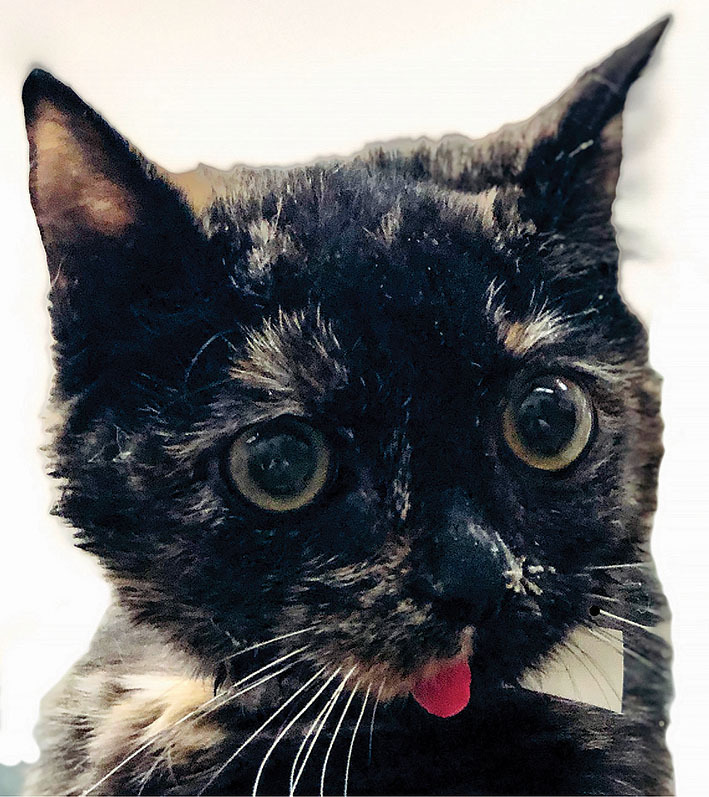
A 6-month-old cat that was presented with tongue entrapment secondary to unilateral TMJ ankylosis. The tongue is trapped in between the mandibular and maxillary canine and incisor teeth.

At initial presentation, all cats had severely restricted mouth opening; six of seven cats had only 2–3 mm of mouth opening from maxillary incisor to mandibular incisor teeth. Cat #5 was noted to have 18 mm of mouth opening, the least restricted of all cats. Two cats (#3 and #6) exhibited severe tongue entrapment, making these cases more urgent in nature due to concerns of upper airway obstruction (Figure 3).

### Preoperative CBCT

**Figure 4:**
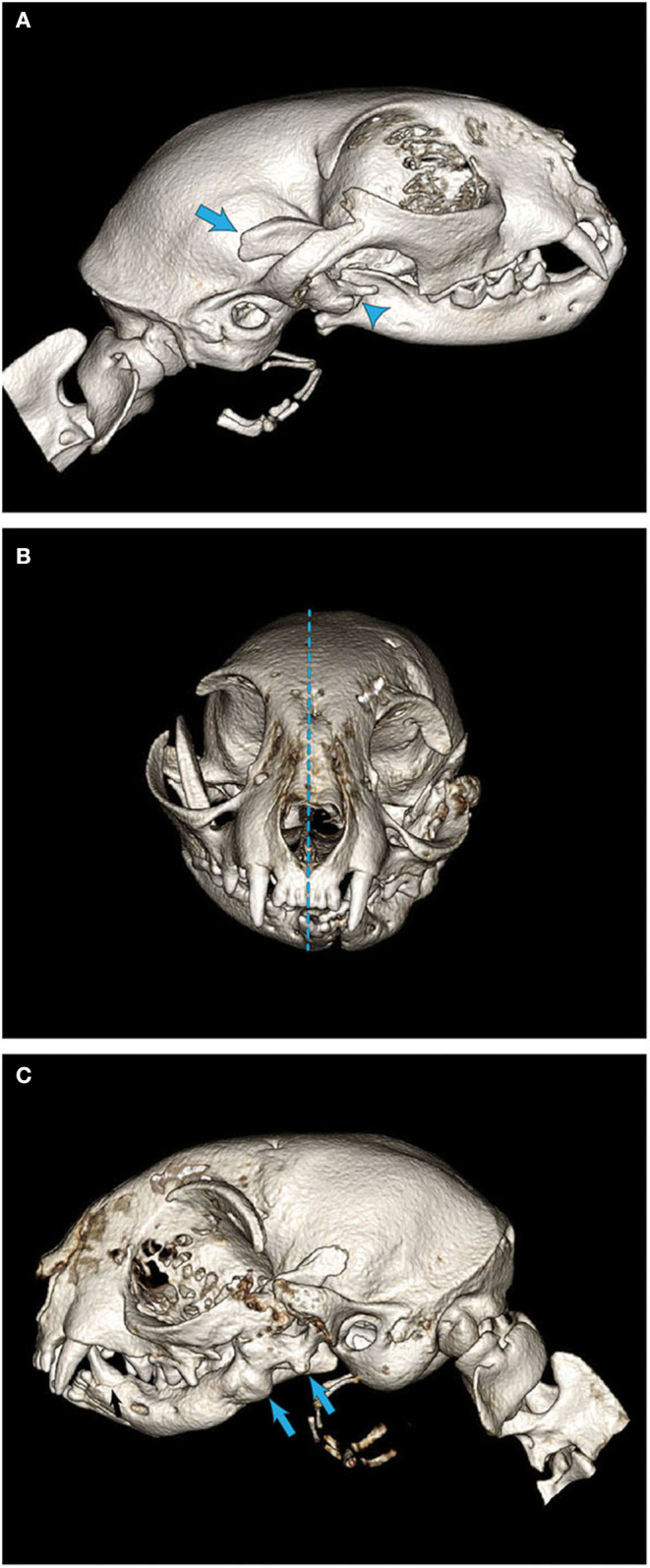
Three-dimensional CBCT examples of skull alterations noted in cats with intra- and extraarticular ankylosis. **(A)** An example of a cat with an elongated and caudally curved coronoid process (arrows). **(B)** Significant asymmetry and malocclusion as demonstrated by the deviation of the mandibular symphysis from midline (dotted line). **(C)** A healed mandibular fracture with bony callus formation (arrows) seen in a cat with historical maxillofacial trauma.

In all seven cats, CBCT confirmed the clinical diagnosis of intraarticular and/or extraarticular TMJ ankylosis. Disease was severe and bilateral in five of the seven cases. In cats #6 and #7, both exhibited unilateral, left-sided TMJ ankylosis. All affected TMJs exhibited joint space narrowing to complete loss of the joint space. Loss of the joint space implied bridging bone across the joint resulting in complete immobility. The additional finding of an elongated and caudally curved coronoid process was documented in the abnormal joints as well (Figure 4A).

Other findings on CBCT included abnormal occlusion in three of the seven cats (a caudal crossbite and mandibular brachygnathism; Figure 4B). Impacted, non-vital, and odontodysplastic teeth were documented in two cats. Additional signs of trauma (previous fractures and a non-union fracture) were documented in two cats (Figure 4C).

### Gap Arthroplasty

In six out of seven cases bilateral gap arthroplasty was performed under one anesthetic episode. One case had staged surgery due to progressive ankylosis of the contralateral side 1 month after the initial surgery. A second case had repeated bilateral gap arthroplasty 6 weeks after the initial surgery due to rapidly progressive intraarticular and extraarticular ankylosis resulting in severely restricted mouth opening. In all cases achieving the surgical goals were measured by the ability to open the patient's mouth without restricted range of motion.

Four cats were presented with pre-existing malocclusions secondary to presumed historic maxillofacial trauma, and two of these cats had the additional finding of impacted and odontodysplastic teeth. Postoperatively, three cats developed a malocclusion resulting in soft tissue trauma to the hard palate that was subsequently treated with tooth extractions.

### Postoperative CBCT

**Figure 5:**
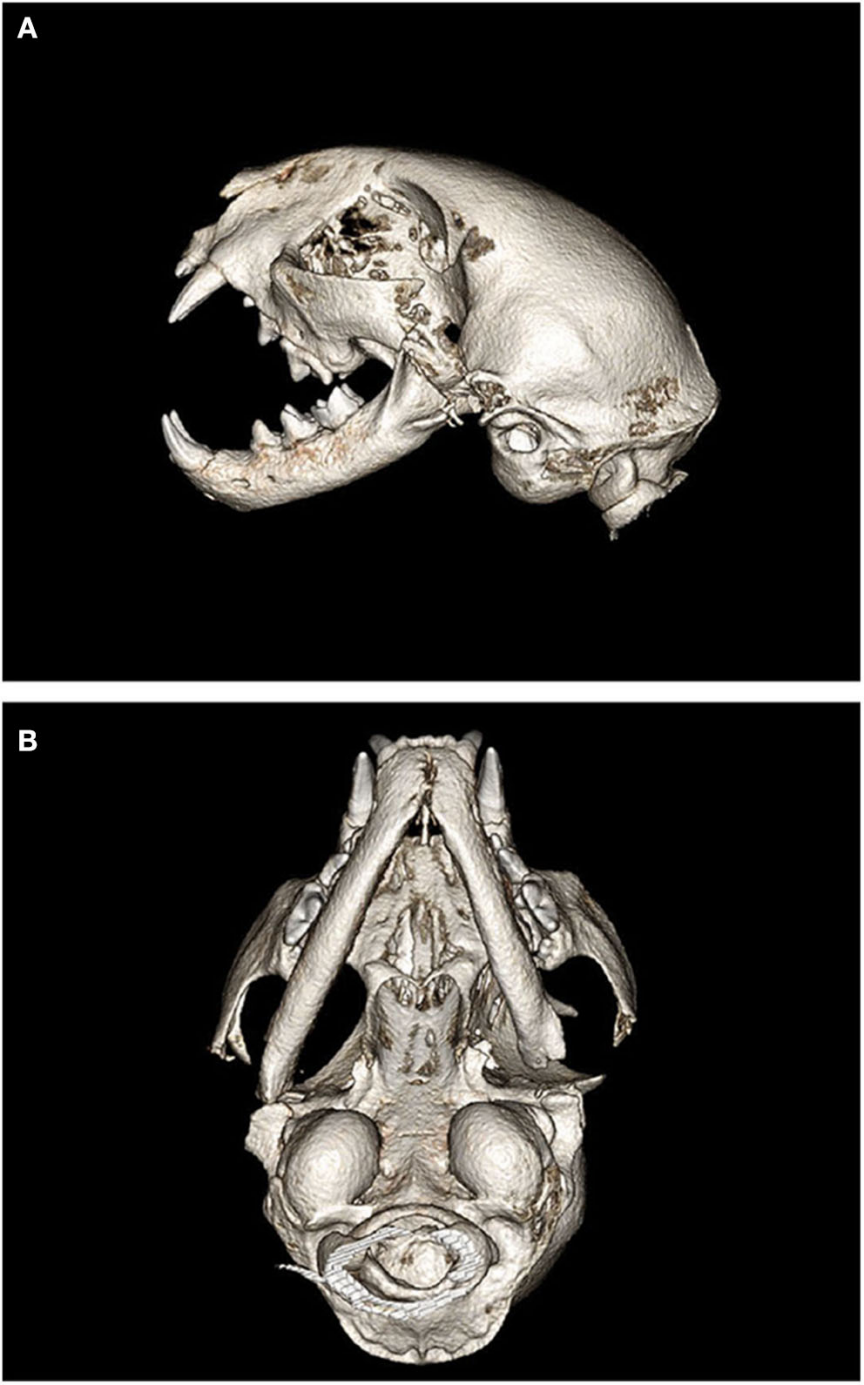
Postoperative 3D CBCT images of cat #7 after bilateral gap arthroplasty. **(A)** Left lateral view depicting the ostectomies of the zygoma, coronoid process, condylar process, and mandibular fossa resulting in a “gap.” **(B)** Ventral view depicting the left sided gap arthroplasty with more conservative ostectomies on the right side.

The results of all postoperative CBCT were interpreted by a board-certified veterinary radiologist along with two experienced board-certified veterinary dentists and oral surgeons. In all cases appropriate ostectomies (zygomectomy, coronoidectomy, condylectomy, and fossectomy) were documented (Figures 5A,B). Additionally, all cases except one had subjectively large enough gaps to prevent contact of the cut ends of bone. The exact location of each ostectomy was dependent on the individual cat, the location, and extent of ankylosis. Postoperative imaging was used to document the ostectomies, and determination of surgical success was based on the clinical ability to open the patient's mouth postoperatively. Gap size was dependent on the extent of ankylosis, patient size, and surrounding anatomic structures.

Immediate postoperative CBCT documented 1 case in which fragments of the zygomatic and retroarticular processes were remnant with a linear osteotomy line at the base of these two structures. The osteotomy line breached partial thickness of the calvarium and a small amount of focal gas accumulation was noted within the calvarium. The cat was monitored closely for any neurological signs postoperatively (none occurred) and the owner was also warned to monitor for any neurologic signs once the cat was discharged from the hospital (none reported). Importantly, all cats ate enthusiastically the same day of surgery. Cats were offered canned food or a slurry and were able to immediately lap up the food without signs of discomfort. One cat was noted to be grooming the day of surgery. All cats were discharged within 24–48 hours.

### Complications

Complications after surgery were minimal. Immediately after anesthetic recovery one cat was noted to have a constricted left pupil, along with exophthalmos, and an incomplete blink in the left eye. Two additional cats recovered and were discharged with an incomplete blink and required topical ophthalmic lubrication. These signs all resolved within 2–3 weeks and were suspected to be secondary to postoperative swelling and inflammation.

### Follow-Up

At the 10–14 day recheck appointment for suture removal, three cats were reported to be thriving at home while the remaining three cats presented with improved but persistent difficulty with prehension of food. The three cats with reported difficulty with prehension were documented to have malocclusions presumably secondary to historic trauma or recent surgical intervention. The follow-up of one cat was performed by the referring veterinarian, the cat was clinically assessed to have no restriction of mouth opening at 3 months postoperatively.

At the 10 day recheck appointment, one cat was presented with mild focal dehiscence of the left surgical incision. It was also noted that this cat had a mild amount of subcutaneous emphysema in the thoracic region due to the recent tracheostomy. The cat was eupneic and the owner was instructed to continue antibiotics for 10-additional days, continue Elizabethan collar usage, and monitor for worsening of subcutaneous emphysema or respiratory distress. The cat continued to recover without complication.

The duration of patient follow-up (including recheck appointments without diagnostic imaging) ranged from 2 to 13 months. Recheck appointments without diagnostic imaging were performed to assess the patient's function (ability to eat, groom, and overall quality of life), as well as to assess the range of motion of the jaws.

Follow-up with recheck CBCT was recommended for all cats, but only performed in three. One cat had a 4 month follow-up CBCT after bilateral surgery along with periodontal treatment with multiple extractions to treat a combination of periodontal disease, endodontal disease, and feline resorptive lesions. CBCT showed a moderate amount of smooth new bone formation along both of the mandibular ostectomy sites growing toward the remaining portion of temporal bone, resulting in bilateral pseudoarthrosis of the TMJs. Despite the findings on imaging, the cat had normal range of motion at that visit and was lost to further follow-up.

**Figure 6:**
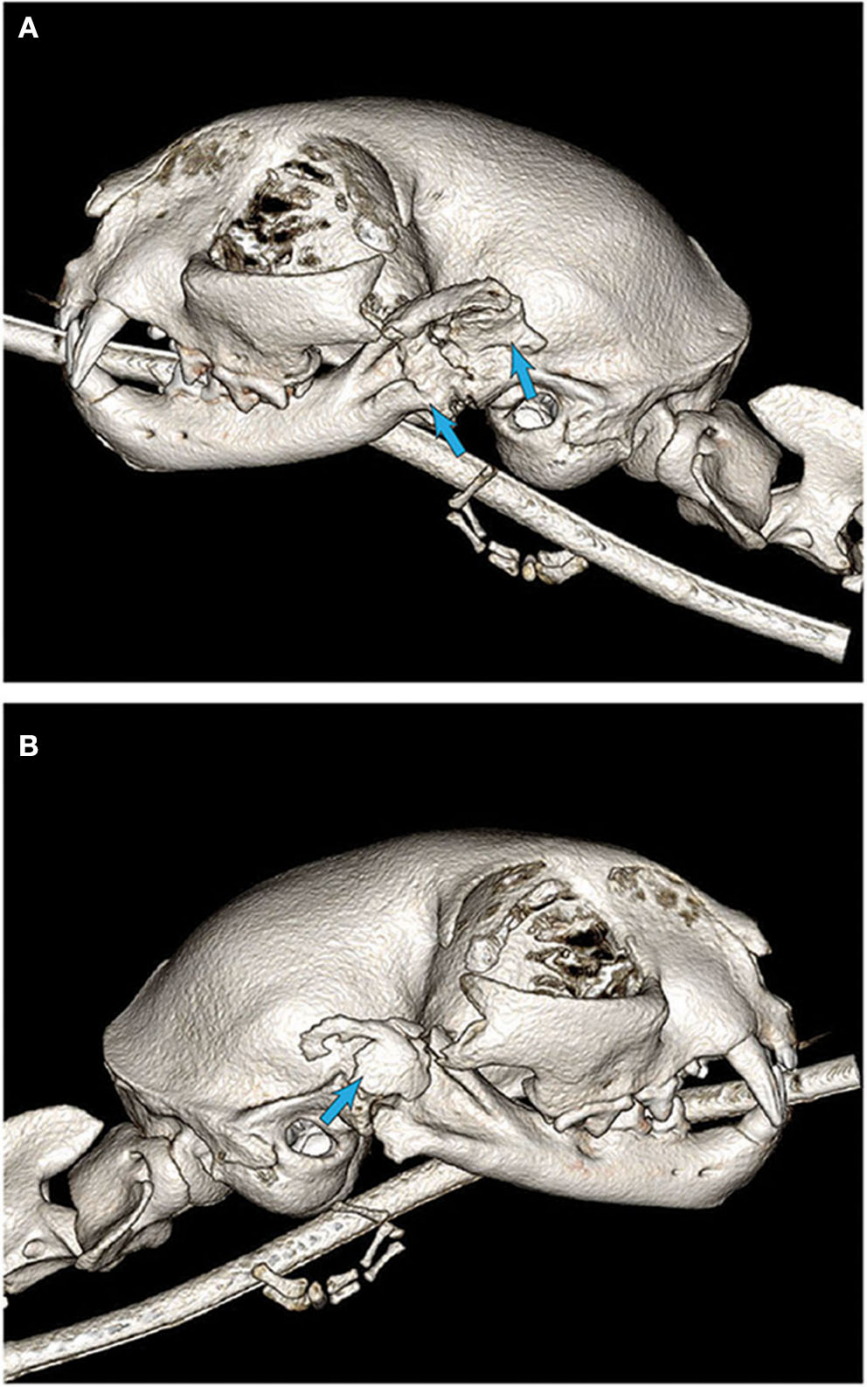
3D CBCT images of cat #7 6 weeks after initial surgery. **(A)** Note the substantial new bone formation stemming from the previous ostectomy sites (arrows). **(B)** New bone formation that is partially organized, at the location of the historically excised coronoid process (arrow).

A second cat was presented 6 weeks after initial bilateral gap arthroplasty with acute onset of restricted mouth opening. On examination the cat was noted to have <10 mm of mouth opening with stable mandibles and loss of a palpable gap bilaterally. This cat underwent general anesthesia for diagnostic imaging with plans for immediate surgical revision of previous gap arthroplasty. This cat required endoscopic-guided endotracheal intubation. CBCT revealed extensive new bone formation bilaterally resulting in pseudoarthrosis and pseudoankylosis (Figures 6A,B). This cat had repeat bilateral gap arthroplasty with routine recovery. At the suture removal appointment 14-days postoperatively, the cat showed ~50% of normal range of motion, which was suspected to be secondary to continued bone formation and muscle fibrosis. Despite restricted mouth opening, the cat was reported to be thriving at home. This cat was rechecked again at 6 weeks and showed continued reduction of range of motion resulting in ~25% of normal mouth opening. Despite this further reduction, the cat was reported to be doing well at home but was no longer able to consume large kibble. The owners were instructed to monitor closely for any further restriction as additional surgical intervention may be required.

The third cat had a 6-month follow-up CBCT after staged bilateral gap arthroplasty. The right TMJ region had a palpable gap with mandibular mobility. The left TMJ had loss of palpable gap and the mandible was stable on palpation. The mouth opening from maxillary to mandibular incisor teeth while awake was restricted to ~25 mm. CBCT demonstrated that the left gap arthroplasty site had formed a pseudoarthrosis with amorphous new bone formation with near loss of the previous gap. The cat was discharged to the owner with instructions to monitor for any further restrictions in mouth opening. If further restriction occurred, surgical revision would be recommended.

## Discussion

To the authors' knowledge, this is the first case-series report of gap arthroplasty performed in cats. This report demonstrates several clinically relevant aspects in cases of intraarticular and extraarticular TMJ ankylosis with subsequent treatment with gap arthroplasty. First, all cats were young with suspected or documented maxillofacial trauma. Second, diagnostic imaging by means of CBCT was essential for diagnosis, surgical planning, and follow-up. Third, the use of 3D printing was essential in precise surgical planning and execution. Fourth, gap arthroplasty is a delicate procedure, requiring precision ostectomy. Finally, dental malocclusion was noted and had a history of variable clinical presentations and consequences.

In all cases, historical maxillofacial trauma at a young age was suspected or known to be the cause of the underlying TMJ ankylosis. In humans, maxillofacial trauma is the leading cause of bony ankylosis of the TMJs (1, 12). TMJ ankylosis can be seen in human pediatric patients due to trauma during the birthing process, and less commonly due to an underlying congenital condition (1, 7, 8, 13). Some authors have suggested that traumatic injury of the TMJ in young human and animal patients can result in increased severity of ankylosis due to the excellent healing capacity noted in younger patients and due to the development of an exuberant bony callus (4, 10, 12). In this report, documented injuries included high-rise falls, dog attacks, and vehicular trauma. Therefore, it is essential that veterinarians and owners are aware that maxillofacial trauma in young cats, especially in the region of the TMJs may result in a life-limiting restriction of mouth opening later in life. Hence, as soon as restricted mouth opening is noted, diagnostic imaging should be performed, and early intervention is recommended.

Due to the size of a cat's skull and complexity of the local anatomy in the region of the TMJs, conventional radiography has been shown to be an inadequate imaging modality (14). Furthermore, conventional radiography is likely to underestimate the extent of ankylosis and true bony involvement. In fact, conventional CT was shown to be superior to skull radiographs when identifying specific anatomical structures of the feline skull (14). Therefore, diagnostic imaging by means of CT or CBCT are considered the gold-standard imaging modalities (1, 3, 4, 6, 10, 14, 21).

In order to enhance the understanding of two-dimensional (2D) imaging, the use of CT and CBCT is recommended with the use of 3D rendering. The use of 3D rendering is essential to allow the clinician to have a thorough understanding of the involved anatomic structures, to document the extent of ankylosis, and to assess the skull and jaws for any other signs of trauma or congenital abnormalities (4, 6, 15, 21). The use of CT or CBCT imaging can be employed to create a 3D printed model of the patient's skull, allowing the surgical team to precisely plan the sites of ostectomy (4, 6, 15). Based on the authors' experience, the use of anatomical landmarks in reference to planned ostectomy sites on the 3D printed model proved to be a valuable resource in the operating room.

In all cases, precision ostectomy has been performed using a piezotome with bone cutting tips. The piezotome was demonstrated to be an effective surgical tool that allows for accurate bone cutting with reduced risk of hemorrhage and damage to surrounding soft tissues and neurovascular structures (6, 19, 20). The piezotome is an innovative surgical instrument that uses high frequency (25–35 kHz) vibration of a metallic tip to selectively cut bone while sparing neurovascular structures and soft tissues (19). Use of a piezosurgical unit also results in more rapid healing (19, 20). The piezotome can also be used to perform osteoplasty with specialized bone surgery tips that allow for smooth shaping and contouring of bone margins. The disadvantages of the piezosurgical unit are that it tends to be slower than traditional oscillating saws or cutting burs and a learning curve exists for the operator of the instrument (19, 20). Regardless, given the delicate nature of the surgery and the challenging anatomic location, the authors recommend the use of a piezotome when performing gap arthroplasty in cats.

In the authors' perspective, gap arthroplasty is considered to be an invasive salvage procedure for cats that have poor quality of life or risk of death secondary to restricted opening of the mouth. Gap arthroplasty allows cats to have immediate mouth opening by removal of all intraarticular and extraarticular structures associated with the ankylosis. All cats were able to return to normal function in terms of prehension of food and water, grooming, and vocalizing within hours of surgery.

Creation of a subjectively appropriate gap is essential for reducing recurrence of ankylosis, along with immediate return to function as physiotherapy to prevent refusion (4, 6, 22, 23). In humans, the three surgical treatment options for TMJ ankylosis include: (1) gap arthroplasty; (2) interpositional arthroplasty; and (3) reconstruction of the articulation (22, 24). Interpositional arthroplasty involves the insertion of an interpositional material after resection of the ankylosis in hopes of preventing re-fusion, while reconstruction of the articulation can occur using autogenous or alloplastic grafts (22, 24). The current strategy in cats is aimed at removing of the ankylotic structures (i.e., salvage procedure) with the intention of immediate return to essential functions such as eating and drinking. Physiotherapy in feline patients can be challenging, therefore immediate return to hard food is recommended to encourage masticatory movement of the jaws. All clients were encouraged to engage in play with the cats postoperatively, specifically encouraging biting/chewing behaviors in an attempt to prevent any further ankylosis. Future options and advancements may become available in veterinary medicine to enhance the functionality of the TMJ.

Dental malocclusions and mandibular instability are expected consequences of gap arthroplasty. Malocclusions may be pre-existing or may develop secondary to surgical treatment. In the authors' experience cats tolerate the mandibular instability well and were typically able to close their mouths using muscle tone within 30 minutes to 4 hours postoperatively. Once cats healed from the initial surgery, regularly timed recheck examinations should reveal if there is a need for tooth extractions to prevent trauma secondary to malocclusion. An additional consideration is that the young cats with TMJ ankylosis seen in this report had more significant periodontal disease as compared to their normal counterparts. Therefore, oral and dental care such as a full periodontal treatment and regular tooth brushing should be initiated after healing from surgery.

Limitations of this study include a small sample size and follow-up time of <2 years in all patients. Lastly, only three of the seven patients had repeated diagnostic imaging performed to assess for recurrence of ankylosis. Therefore, the follow-up of the remaining four cats was based on clinical assessment of mouth opening and performing basic functions such as eating, drinking, and grooming.

In summary, gap arthroplasty is an invasive and delicate procedure that allows for immediate return to function. Proper surgical imaging and preparation are essential for a positive surgical outcome resulting in a cat that can have functional mouth opening. The downfall of gap arthroplasty is that despite extensive removal of bone and creation of a gap, there is a possibility of varying degrees of recurrence of ankylosis as demonstrated by two cats in this report. However, the risk of the surgery is outweighed by the alternative of euthanasia or deterioration of health and subsequent death for these cats. The immediate return to function allows for the restoration of acceptable quality of life as demonstrated in these cats by the immediate ability to eat, drink, and groom.

## Data Availability Statement

The raw data supporting the conclusions of this article will be made available by the authors, without undue reservation.

## Ethics Statement

Ethical review and approval was not required for the animal study because the study is retrospective in nature and included clinical cases, hence, it is exempt from IACUC requirements. The standard written informed consent required for all procedures performed at the William R. Pritchard Veterinary Medical Teaching Hospital of the University of California, Davis was obtained from the owners. Written informed consent was obtained from the owners for the participation of their animals in this study.

## Author Contributions

AA: study concept and design, provision of study material or cases, manuscript writing, data analysis, and interpretation. BA and FV: study concept and design, provision of study material or cases, manuscript writing, data analysis and interpretation, and review of manuscript for important intellectual input. All authors contributed to the article and approved the submitted version.

## Conflict of Interest

The authors declare that the research was conducted in the absence of any commercial or financial relationships that could be construed as a potential conflict of interest.
